# Overcoming the barriers to implementation of competence-based medical education in post-graduate medical education: a narrative literature review

**DOI:** 10.1080/10872981.2022.2112012

**Published:** 2022-08-12

**Authors:** Jayson M. Stoffman

**Affiliations:** Department of Pediatrics and Child Health, Director, Pediatric Postgraduate Medical Education, University of Manitoba, Winnipeg, MB, Canada

**Keywords:** competence-based medical education, faculty development, resident education

## Abstract

To ensure that residents are equipped with the necessary skills for practice, competence-based medical education (CBME) represents a transformative change in postgraduate medical education, which is being progressively introduced across Canadian specialty residency programs. Successful implementation will require adjustments to curriculum, assessment, and evaluation, with careful attention to the unique needs in the local context, including resident and faculty development. This narrative review of the literature aimed to determine the potential barriers to the successful implementation of CBME and the strategies by which they can be addressed, with a specific consideration of the author’s program in pediatrics in Manitoba. Eleven articles were identified with a specific focus on the implementation of CBME in the post-graduate setting, and 10 were included in the review after critical appraisal. Three key themes emerged from the articles: the value of broad stakeholder engagement and leadership, the importance of faculty and resident development, and the development of specific support systems for the educational curriculum. Different strategies were considered and contrasted for addressing these important themes. This review provides important insights and practical approaches to the barriers that should be useful as programs prepare for the implementation of CBME.

## Background

In light of the increasing complexity of health care and the importance of social accountability in its delivery, a competency-based approach to medical education is meant to ensure that recently graduated doctors, generally called residents, are equipped with the necessary skills for their ultimate specialty practice [1]. Competence-based medical education (CBME) is a shift from the traditional apprenticeship model to outcomes-based training, where the ultimate goal is for trainees to develop and demonstrate the necessary competence for patient care [2–4]. Dagnone et al. [5] describe the introduction of CBME as a transformative change for the medical education community, which will necessitate an ‘evidence-based way of thinking and acting to both prepare our community … and support us through emergent adaptations’ (p. 1644). In Canada, the Royal College of Physicians and Surgeons is responsible for the oversight of training, evaluation, and certification of medical and surgical specialists, and is currently in the process of progressively introducing a competency-based approach in all its post-graduate medical education programs [6].

In his commentary on outcomes-based medical education, Holmboe [7] describes that CBME provides opportunities for all learners to go beyond basic medical knowledge and clinical skills to emphasize competencies in the core activities of the discipline. Application of these competencies is enabled through the definition of Entrustable Professional Activities (EPAs), which are the specialty-specific tasks which the resident is delegated to perform independently once competence has been demonstrated to their supervisor [6]. Each EPA includes several milestones, which represent the measurable and developmental indicators of the resident’s ability to perform the task [2]. Documentation of progress on milestones and EPAs requires a shift from global evaluations of performance on a clinical rotation and summative examinations to workplace-based assessments in the clinical environment [7].

It is evident that these major cultural shifts in post-graduate medical education will require widespread stakeholder engagement, consideration of interpersonal and group dynamics, and careful evaluation of implementation needs in the local context [5]. In preparing to introduce CBME into the anesthesiology residency program at the University of Ottawa, Stodel et al. [2] describe the importance of using a resident-centered approach in their curricular design, with an emphasis on active learning and individualized progression through the program as measured in evidence-based milestones and EPA. They also describe the need for a comprehensive system of assessment and evaluation, and the importance of faculty development and change management for both residents and faculty in transitioning to this new educational paradigm.

Pediatric training in Canada is scheduled to implement CBME with the residency cohort starting July 2021. As the program director for pediatrics at the University of Manitoba, the author will be directly responsible for leading this transition in our program, with 40 residents and a faculty of nearly 120 physicians. Drawing on the experiences of other programs, the aim of this narrative review is to determine the potential barriers to the successful implementation of CBME in pediatrics in Manitoba and the strategies by which they can be addressed.

## Methods

This literature review identified scholarly research in CBME through the electronic databases available at the author’s university library (www.twu.ca/library). Databases included in this review were the university library’s OneSearch, ERIC (Education Resource Information Center), MEDLINE, Academic Search Ultimate, and CINAHL. The following key subject terms were used to search the databases with Boolean ‘AND’ to combine the search terms, limited to English language: ‘competenc*-based medical education’, post-graduate OR residen*, transition OR change. This search strategy was designed deliberately to focus on those papers with a focus on change management efforts in the introduction of CBME to PGME programs. After removing duplicate results from the different databases, a total of 80 papers were identified. Those articles were then manually screened by title and abstract, and 15 articles were identified as potentially relevant to this review. The same search was performed on Google Scholar which did not result in any new results. It is important to note that the search parameters did not limit results by region or country, although this could be implicit in the English language limitation.

### Inclusion and exclusion criteria

The 15 articles were either retained or eliminated based on the inclusion and exclusion criteria. All articles were evaluated by the solitary author (JMS) according to criteria established a priori for this narrative review. The reviewed articles were required to meet the following criteria: 1) peer-reviewed papers published between 2011 and 2021, 2) accessible through online sources, and 3) implementation of post-graduate CBME as a central theme. Conversely, articles were excluded based on the following criteria: 1) focus on undergraduate medical education, independent practice, or continuing professional development, or 2) health-care transformation as the central theme. Of the 15 articles identified, 11 articles met the inclusion criteria and were considered in the review. These 11 articles were drawn from a variety of scholarly journals.

### Critical Analysis

Each of the 11 articles was critically appraised using the methods described by Plano-Clark and Creswell [8], for quantitative or qualitative research methods as appropriate. Essentially, this entails an assessment of each section of the research paper [Introduction, Methods, Results, Discussion/Conclusion] using specific criteria relevant to the section contents and general criteria evaluating the contribution of the section to the overall research paper. The majority of articles identified were of adequate or high quality and included in the review. One paper, an invited commentary by Dagnone et al. [5], was excluded at this stage, while it did provide an interesting perspective on the high-level priorities of CBME, it did not contribute in a relevant way to the purposes of this review.

## Results

The articles included in this review represent a variety of perspectives and approaches to the challenges in implementing CBME within post-graduate medical training programs. Three articles provided a broad systems review [9–11]. Three were surveys looking at perceptions of faculty and residents [12], faculty needs assessment [13], and program preparedness [14]. Two studies used qualitative interviews to explore perceptions of residents [15] and both residents and faculty [16]. Finally, there were two case studies of a single program, one in the emergency department [17] and one in anesthesiology [18]. Across these different articles, three important themes emerged: the value of broad stakeholder engagement and leadership, the importance of faculty and resident development, and the development of specific support systems for the educational curriculum.

### Stakeholder engagement and leadership

Strong leadership is needed to champion the changes of CBME, which requires program leaders ‘to engage in a community of shared learning and leadership’ [10, p. e32]. The health-care curriculum should be integrated with health human resources planning with educational continuity realized as ‘policies that support the principles of CBME at all levels of medical education’ [11, p. 594], from the early undergraduate trainee to the continuing professional development of the practicing physician. The literature alone is limited in disseminating knowledge and best practices of CBME, and a community of practice to share information both within and between programs has significant advantages [16]. The Canadian Pediatric Program Directors group is a specific example of how this has been fostered in the author’s own discipline.

Another frequently raised concern was commitment and engagement from the faculty and residents directly impacted by CBME [15,16,18]. Beyond leadership, centralized institutional governance and support for CBME, with the appropriate allocation of financial support, time, and educational resources can improve engagement and participation [10,18]. From their interviews with residents, Mann et al. [15] called for increased opportunities for resident engagement and feedback on the implementation of CBME, which were ‘repeatedly suggested as vital to a successful transition’ (p. e37). This was supported in the systems-based review by Dagnone et al. [10], who described the value of establishing a resident sub-committee at their center to provide a forum for resident advocacy and discussion.

Ultimately, stakeholder engagement needs to include and be driven by the institutional leadership and priorities, which may be challenging to affect in that they are often external to the program itself [18]. Returning to the concept of educational continuity, clinical program redevelopment should occur in concert with the educational redesign of CBME, requiring the consideration of potential medical regulatory changes and input of those bodies, with mutual accountability of the different stakeholders throughout the implementation process [9]. This was validated by Hall et al. [17], in their evaluation of CBME implementation in a single emergency medicine program; ongoing efforts at continuous quality improvement led to better investment in the process by the different stakeholders.

### Faculty and resident development

Fraser et al. [18], define faculty development as ‘any planned activities designed to improve an individual’s knowledge, skills, and attitude in areas related to the roles and responsibilities of a faculty member’ (p. 1365), and this definition applies equally to resident development. Their article provided a review of initiatives undertaken in their anesthesiology program at the University of Ottawa; they initially found a lack of faculty knowledge and skills to shift to CBME, a lack of technological expertise for delivery of CBME curriculum, and a lack of time and ability to provide proper resident assessment. Other studies also noted the importance of defining key terminology in CBME, particularly in relation to frequency of observations and assessment and criteria for entrustment ratings [9,16].

Stefan et al. [13] uncovered similar issues in their more detailed and structured needs assessment of Canadian emergency medicine program faculty and trainees. They describe that their faculty generally believe they give good feedback to residents but have a desire to improve. These faculties identified the time required to deliver workplace-based observations, potentially impacting patient flow and care, and the possible repercussions of delivering negative feedback as barriers to effective assessment. Echoing the need for clear definitions, their survey also uncovered a general lack of familiarity with the details of CBME, which was their explanation for the finding that nearly half of respondents did not anticipate benefits in training or patient care with CBME. Similar concerns were identified in medical oncology programs [14].

These observations highlight the need for faculty development programs, addressing the curricular format of CBME and the process of workplace-based assessment [18]. In their interviews with residents, Mann et al. [15] identified the need for appropriate faculty development to create a change in feedback culture, which Caverzagie et al. [9] also describe as a requirement for CBME to be successful. Hall et al. [17] observed that similar culture change efforts within the emergency medicine program led to ‘substantial efforts and adaptations in the form of faculty and resident development activities relating to the provision, documentation, and acceptance of constructive feedback’ (p. 790). They describe this as a core component of CBME, requiring iterative cycles of instruction, practice, and feedback to fully develop, consistent with their discussion of the value of continuous quality improvement, as presented in the first theme.

Crawford et al. [12] surveyed both residents and program directors regarding both groups’ perceptions and barriers of CBME, highlighting the importance of both resident and faculty development in the implementation of CBME and the role of the resident in partnering with faculty in their assessments. Their survey found that residents had the least positive opinion of CBME and noted that residents might not recognize the practical relevance of the EPA, since they are not yet fully versed in the skills of clinical practice. Their call for residents to be engaged in CBME leadership echoes the call in the first theme for communication and engagement with residents throughout the implementation of CBME [10, 15]. In the author’s own program, a dedicated workshop has been introduced for all first-year residents in the first weeks of training to set a strong foundation for their experience of CBME.

### Educational and technological support

The resident-focused approach to training was highlighted in several articles [10,11,14–16]. Nousiainen et al. [11] refer specifically to the use of time as a resource rather than an endpoint in CBME, necessitating the changed approach to health-care education as described in the first theme. While recognizing the value of personalizing a resident’s training according to their own developmental needs, Boet et al. [16] listed potential adverse impacts, such as a desire to rush through training to enter the workforce at full salary, competition for training experiences and observations, and the administrative challenges of individualizing training programs. In their interviews, Mann et al. [15] also observed that such practical and logistical challenges were associated with pessimism from the residents regarding CBME, and they advocated for resident input in mitigating these challenges.

In addition to the administrative reorganization of training experiences, appropriate curriculum delivery, assessment, and evaluation will also require educational and technological support [11,12]. Simulation may be necessary for those EPA that are less frequent [10], and Arora et al. [14] describe how this might actually create new training opportunities. In the author’s program, this has led to the creation of a robust simulation curriculum to address a variety of EPA and competencies.

These core pedagogical elements require rapid introduction in the early implementation of CBME, to provide appropriate systems, support, and leadership as foundations for the subsequent faculty and resident development [17]. Solutions to these practical challenges are suggested in the previous two themes, with stakeholder engagement, strong leadership, and faculty and resident development playing important roles. Of course, the specific solutions are dependent on the unique institutional context of the programs, as illustrated in the examples provided above.

## Discussion

In exploring the barriers to implementation of CBME and how to address them, this literature review highlights three key themes: the importance of stakeholder engagement and leadership, the value of faculty and resident development for the specific tasks and requirements of CBME, and the implementation of the necessary educational and technological solutions for this new training paradigm. These themes are similar to the high-level priorities identified by Dagnone et al. [5], while their paper did not meet the criteria to be included in this review, their perspective echoes the findings here.

It is reassuring that the barriers and solutions described were consistent across the different articles and research types, suggesting saturation in the focused literature search. From a practical perspective, it is also important to note that the findings of the broad systems reviews [9–11] were echoed in the studies of specific programs [13,14,16–18]. While the themes identified here could be more broadly applied to any change in medical education, this suggests real-world utility of the proposed solutions in a post-graduate setting.

One limitation of the review is the generalizability of the results. Many articles reflected the perspectives and findings of a single residency program [13,14,16] and often at a single site [17,18]. There were also challenges with the variety of methodologies, and methodologic rigour, used by the individual articles. The two case studies provide useful information, but the review process is unclear in Fraser et al. [18], and not validated by observation in Hall et al. [17]. Mann et al. [15] used a grounded theory approach in their semi-structured interviews with residents, while Boet et al. [16] failed to achieve saturation due to poor recruitment. The commonality of findings suggests that these limitations did not ultimately impact the validity of the identified themes, but a future narrative or systematic review could broaden the search strategy to capture other programs and regions.

For future evaluation of CBME implementation, action research will allow iterative reviews as knowledge and experience is gained to highlight those barriers that are most significant and suggest implementation strategies to overcome them. Action research can look specifically at the impact of the local training program context on the barriers to implementation and their solutions, identifying the technological and human resources that are most applicable in that program and more broadly. Further qualitative studies will also be valuable to assess the changing perceptions and impact of CBME over time and may identify previously unexpected or unappreciated challenges that will need to be addressed. This is analogous to the continuous quality improvement advocated by Hall et al. [17].

## Conclusion

As pediatrics training programs across Canada prepare to implement CBME, we are collaborating on strategies through our national program directors’ working group. This has been a valuable community of practice to share experiences and has already influenced our faculty, resident, and curricular development efforts. While the opening perspective of this narrative review was the implementation of CBME in a single postgraduate medical education program, the combined experience reviewed herein bears informative witness to the common challenges. It will also be valuable to capture the undergraduate medical education experience of CBME to add to the depth and breadth of the identified themes.

This narrative review is meant to provide an introduction to the challenges in the implementation of CBME and to inform future efforts by medical education programs and communities of practice. The three identified themes provide important insights and practical approaches to the barriers that we are likely to encounter. Identifying and understanding them will benefit the Manitoba pediatrics program in our transition, and our experience will add to this knowledge for those programs who have yet to move to CBME.

## Authors’ information

JMS is the Director of Post-Graduate Medical Education for the Department of Pediatrics and Child Health at the University of Manitoba, which recently implemented competence-based medical education for its residency training.
